# Central venous pressure dynamics in neonates with hypoxic-ischemic encephalopathy: insights into high mean airway pressure, PPHN, and ECMO management

**DOI:** 10.3389/fped.2025.1611740

**Published:** 2025-09-11

**Authors:** Tomonori Kurimoto, Tokuhisa Takuya, Masaya Kibe, Toshio Harumatsu, Hiroshi Ohashi, Tsuyoshi Yamamoto, Eiji Hirakawa

**Affiliations:** Department of Neonatology, Perinatal Medical Center, Kagoshima City Hospital, Kagoshima, Japan

**Keywords:** central venous pressure (CVP), hypoxic-ischemic encephalopathy (HIE), persistent pulmonary hypertension of the newborn (PPHN), high mean airway pressure (MAP), extracorporeal membrane oxygenation (ECMO), inhaled nitric oxide (iNO), neonatal hemodynamics, umbilical venous catheter (UVC)

## Abstract

**Introduction:**

Central venous pressure (CVP) monitoring provides valuable insights into hemodynamic changes; however, its application in neonates with hypoxic-ischemic encephalopathy (HIE) undergoing advanced therapies remains underexplored. This study aimed to evaluate the dynamics of CVP under varying conditions, including high mean airway pressure (MAP), persistent pulmonary hypertension of the newborn (PPHN), and treatment with inhaled nitric oxide (iNO) and extracorporeal membrane oxygenation (ECMO).

**Methods:**

This retrospective study included 18 neonates diagnosed with HIE, who received brain hypothermia therapy and had umbilical venous catheters (UVC) appropriately placed for CVP monitoring. CVP values were analyzed in relation to high MAP (≥10 cmH₂O), PPHN status, and pre- and post-therapeutic interventions such as iNO and ECMO. Statistical comparisons were performed using Mann–Whitney *U* tests for continuous variables, with significance set at *p* < 0.05.

**Results:**

Neonates in the high MAP group exhibited significantly higher mean CVP values than those in the normal MAP group (6 vs. 5 mmHg, *p* = 0.03). In the PPHN + high MAP group, the mean CVP, oxygenation index, and FiO₂ levels were markedly elevated compared with the high MAP group without PPHN. iNO administration significantly reduced the mean CVP (7 mmHg pre-iNO vs. 4 mmHg post-iNO, *p* = 0.04), whereas VV-ECMO initiation resulted in an increased CVP (mean CVP: 8 mmHg pre-ECMO vs. 13 mmHg post-ECMO, *p* = 0.03).

**Discussion:**

CVP monitoring via UVC provides critical information on hemodynamic changes in neonates with HIE, particularly under high MAP and PPHN conditions. While iNO effectively reduced CVP and improved oxygenation, VV-ECMO led to elevated CVP, likely due to the return cannula flow. These findings underscore the need for optimized cannula placement and ventilatory strategies to minimize hemodynamic instability during advanced neonatal therapy.

## 1 Introduction

Central venous pressure (CVP) is influenced by four main factors: vis a tergo, the energy transmitted to the venous system by the left ventricular pressure through the capillary bed; vis a fronte, the energy generated by the pumping action of the right heart to return blood from the venous system to the heart; vis a latere, external forces acting perpendicularly on veins such as venous compliance, vascular tone, tissue pressure, and changes in intrathoracic or intra-abdominal pressure associated with respiration; and vis a parte interiore, the energy determined by the relationship between venous blood volume and venous capacity (1).

CVP can be measured in neonates using an umbilical venous catheter (UVC), which is typically used in cases requiring intensive monitoring. Although CVP monitoring provides valuable hemodynamic insights, its role in managing neonates with hypoxic-ischemic encephalopathy (HIE) undergoing advanced therapies, such as inhaled nitric oxide (iNO) or extracorporeal membrane oxygenation (ECMO), has not been thoroughly investigated. This study examined neonates with HIE, who underwent UVC placement and ventilatory support, to evaluate the utility of CVP monitoring. We examined cases in which the catheter tip was appropriately positioned to enable reliable CVP measurement. A comparative analysis of CVP values was performed to assess their clinical relevance and implications. Specifically, CVP measurements were compared among neonates with high mean airway pressure (MAP) vs. those with normal MAP, those with high mean arterial pressure with or without persistent pulmonary hypertension of the newborn (PPHN), and those before and after treatments, such as iNO and ECMO, in cases with PPHN.

## 2 Methods

### 2.1 Study design and patient selection

This was a retrospective observational study conducted at the Neonatal Intensive Care Unit (NICU) of Kagoshima City Hospital, Japan, including neonates admitted between January 2000 and October 2022. Eligible neonates were those diagnosed with HIE who received therapeutic hypothermia and underwent umbilical venous catheter (UVC) placement with appropriate tip positioning for CVP monitoring as part of routine clinical care (2–4). Although informed consent for the use of clinical data was obtained prospectively as part of institutional policy, the research hypothesis and analytical framework were developed retrospectively. Therefore, this study does not conform to a classical case–control design. Instead, the primary objective was to descriptively evaluate CVP dynamics under specific clinical conditions (e.g., high MAP, presence of PPHN, administration of iNO or ECMO), without predefined case or control assignments. This study was approved by the Ethics Committee of Kagoshima City Hospital (Approval No. 2024-06) and conducted in accordance with the Declaration of Helsinki. All included neonates completed therapeutic hypothermia and were discharged.

### 2.2 Inclusion criteria

This study included term or near-term neonates (gestational age ≥ 36 weeks) who were diagnosed with HIE and underwent therapeutic hypothermia in accordance with institutional protocols. All patients had UVCs placed with radiographically confirmed tip positions at the junction of the inferior vena cava and right atrium (T7–T9 level), and CVP was continuously monitored as part of standard intensive care. Patients were excluded if they had major congenital anomalies (such as congenital diaphragmatic hernia, abdominal wall defects, or hydrops fetalis), structural heart disease confirmed by echocardiography, positive blood culture at admission, or if the UVC tip was not appropriately positioned for accurate CVP measurement. Eighteen neonates met the inclusion criteria and were enrolled in the study. All patients required respiratory support and completed therapeutic hypothermia. PPHN was diagnosed based on clinical symptoms and echocardiographic evidence of elevated pulmonary arterial pressure and septal flattening, after ruling out structural heart disease (5). The classification of patients into high and normal MAP groups, and the presence or absence of PPHN, were analyzed retrospectively based on clinical and ventilatory parameters collected during hospitalization (6, 7).

### 2.3 Catheterization and monitoring

The UVC was introduced via the umbilical vein, then navigated through the left portal vein and ductus venosus, passing either the middle or left hepatic vein before ultimately entering the inferior vena cava (IVC) and terminating in the right atrium. The ideal catheter tip position was confirmed by radiography to be at the junction of the IVC and the right atrium, typically between the T7 and T9 vertebral levels (8). Only those patients with appropriate UVC positioning were included in the study. In full-term neonates with sufficient vessel caliber, a DLP aortic cannula (Medtronic, Minneapolis, MN), originally designed for arterial use, was inserted cranially into the right internal jugular vein and used solely as a drainage cannula for VV-ECMO. The cannula's size was well-suited to the vessel diameter and allowed stable drainage flow. Additionally, a double-lumen catheter (Nipro Corporation, Osaka, Japan) was inserted into the right internal jugular vein and advanced into the internal jugular sinus, serving as both the drainage and return routes. CVP was measured after the VV-ECMO pump flow rate was set to 20 ml/kg/min, as this low flow minimized circuit-related suction effects and allowed for a more physiological and comparable measurement of central venous pressure across patients.

### 2.4 Respiratory management

All neonates were intubated and managed with mechanical ventilation. Initial ventilatory support was provided using the synchronized intermittent mandatory ventilation (SIMV) mode. Respiratory acidosis, defined as arterial blood gas findings of pH < 7.2 and PaCO2 > 65 mmHg, persisted despite SIMV settings with a peak inspiratory pressure of 20–21 cmH_2_O, positive end-expiratory pressure of 6 cmH_2_O, and a respiratory rate of 60–65 breaths/min. If these settings failed to reduce PaCO2 by >10% or FiO2 by >20% within 1 h, the mode was switched to rescue high-frequency oscillatory ventilation (9, 10). In high-frequency oscillatory ventilation, the MAP was initiated at 2–3 cmH_2_O above the SIMV setting. The oscillation frequency was set at 12–15 Hz, with an inspiration-to-expiration ratio of 1:1. If adequate PaCO2 clearance could not be achieved at the maximum amplitude, the frequency was reduced to 10 Hz (11). Adjustments were made as needed to optimize CO2 clearance. A high MAP was defined as ≥10 cmH_2_O.

### 2.5 CVP measurement

CVP was continuously measured using a bedside monitor (CSM-1901; Life Scope G; Nihon Kohden, Tokyo, Japan). The collected data included heart rate (HR) and systolic, mean, and diastolic blood pressure, along with CVP values. Measurements were recorded by continuous monitoring 2–3 h after catheter placement. The mean CVP value was calculated as the average of all continuously recorded CVP data points collected at 1-minute intervals over a 2–3 h monitoring period following catheter placement. CVP values were continuously monitored before and 1 h after initiation of iNO or VV-ECMO. All neonates were monitored using standardized ventilatory and sedation protocols to minimize variability in CVP measurements.

### 2.6 Statistical analysis

Categorical variables were analyzed using the chi-square or Fisher's exact test, as appropriate. For continuous variables, nonparametric tests were used based on the type of comparison: the Mann–Whitney *U* test was applied to compare two independent groups, while the Wilcoxon signed-rank test was used for paired comparisons, such as pre- and post-iNO or ECMO initiation. Normality was assessed using the Shapiro–Wilk test, which is more appropriate for small sample sizes. All variables deviated from a normal distribution; therefore, nonparametric tests were applied. Statistical significance was set at *p* < 0.05 with a 95% confidence interval.

### 2.7 Ethical considerations

The UVC used in this study was included as part of routine clinical care and not specifically for research purposes. The study protocol was approved by the institutional review board of Kagoshima City Hospital and was conducted in accordance with the Declaration of Helsinki and the National Guidelines for Human Research Ethics.

## 3 Results

**Figure 1:**
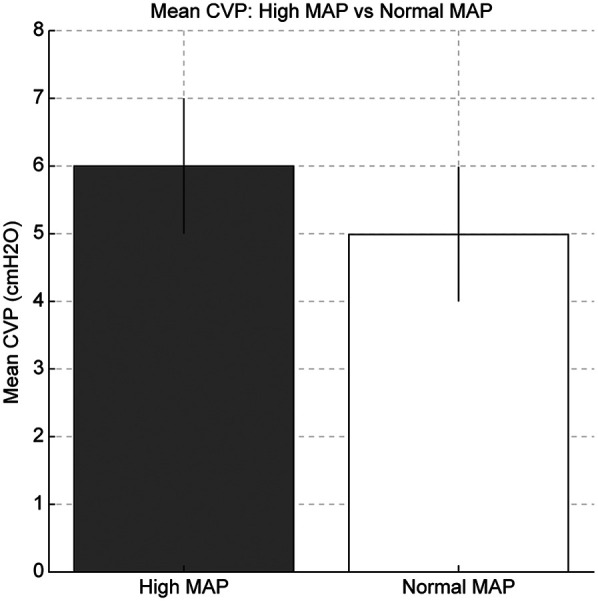
Comparison of oxygenation index (OI) and mean central venous pressure (CVP) between the high and normal mean airway pressure (MAP) groups. The high MAP group showed significantly higher OI and mean CVP, indicating increased intrathoracic pressure due to ventilator setting.

**Figure 2:**
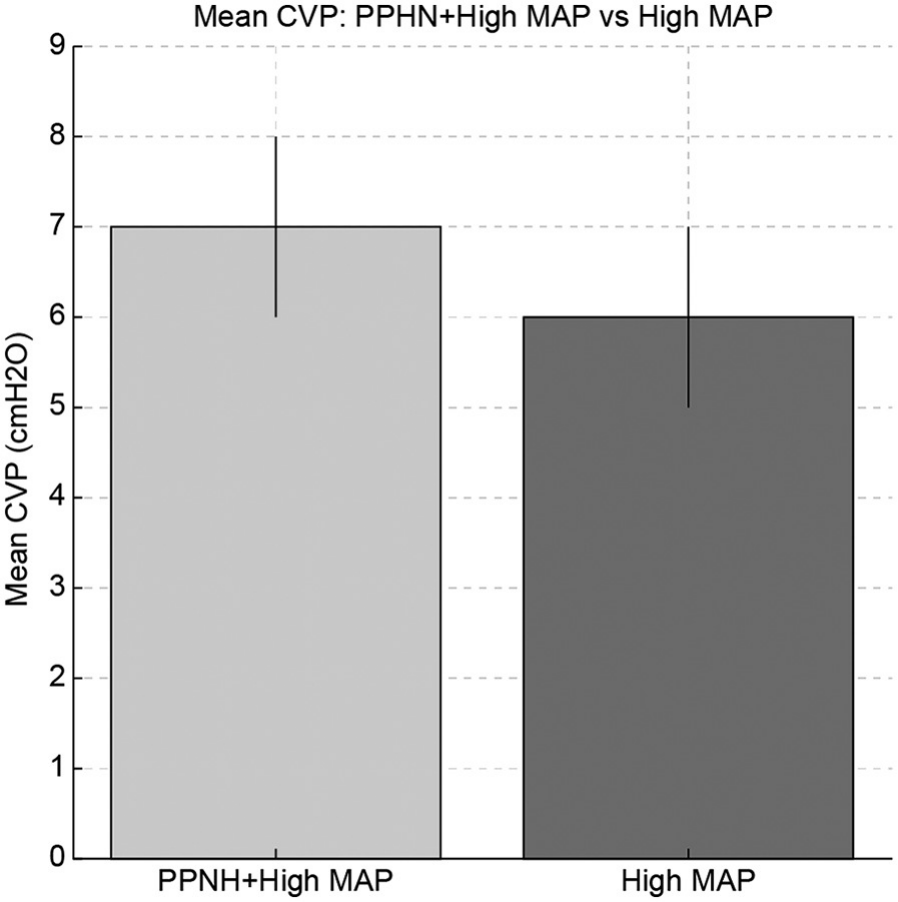
Comparison of mean central venous pressure (CVP), oxygenation index (OI), and fraction of inspired oxygen (FiO₂) between the persistent pulmonary hypertension of newborn (PPHN) + high mean airway pressure (MAP) group and the high MAP group without PPHN. The PPHN + high MAP group showed significantly higher values in all parameters, indicating more severe pulmonary hypertension and impaired oxygenation.

**Figure 3:**
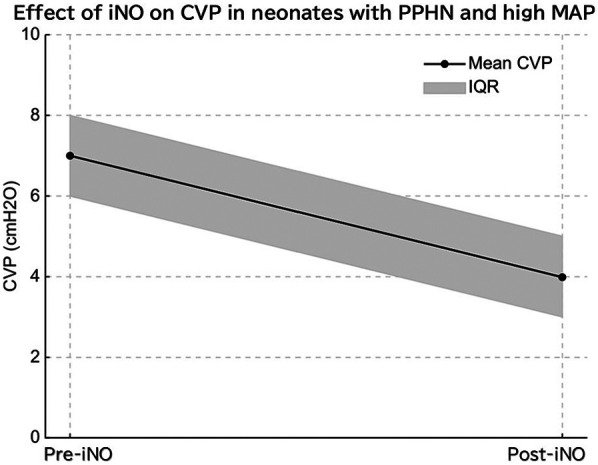
In infants with persistent pulmonary hypertension (PPHN) and elevated mean airway pressure (MAP), inhaled nitric oxide (iNO) administration significantly reduced mean central venous pressure (CVP), indicating decreased pulmonary vascular resistance and improved right ventricular function.

**Figure 4:**
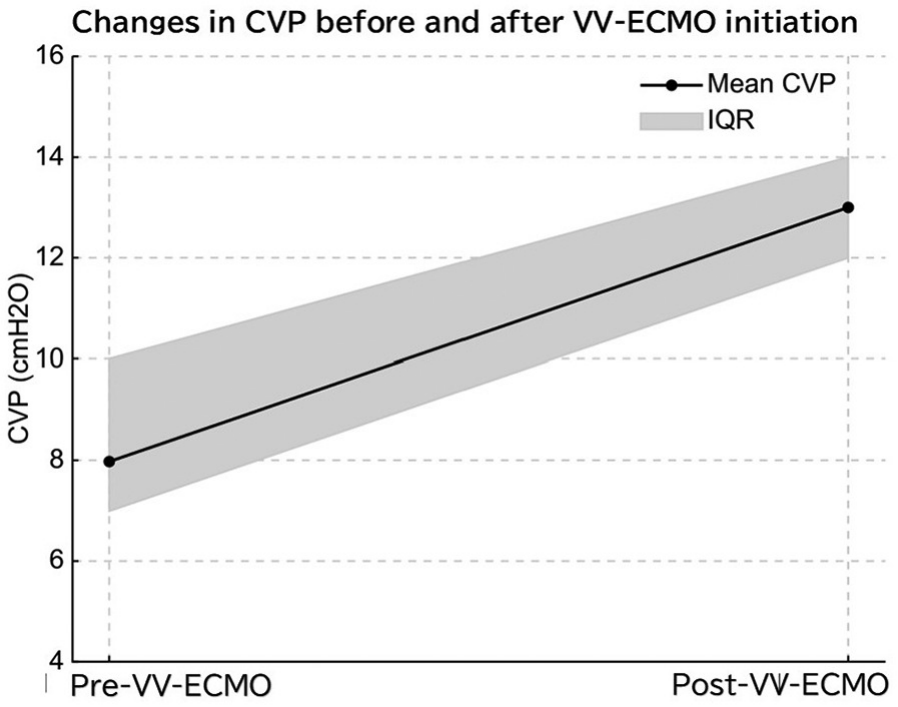
Initiation of venovenous extracorporeal membrane oxygenation (VV-ECMO) led to a significant increase in central venous pressure (CVP), likely due to elevated right atrial pressure from cannula flow return.

Among the included patients, eight had a MA*P* < 10 cmH₂O (normal MAP group), three had MAP ≥ 10 cmH₂O without PPHN (high MAP group), and seven had both high MAP and PPHN. Of the seven patients with high MAP and PPHN, three with an oxygenation index (OI) between 20 and 25 received iNO therapy, while the remaining four with an OI between 25 and 40 underwent veno-venous extracorporeal membrane oxygenation (VV-ECMO) (6, 7). The high MAP group demonstrated significantly higher OI compared with the normal MAP group [median OI: 12.9 (IQR: 5.8–14.2) vs. 2.1 (IQR: 1.8–3.7), *p* = 0.01; Table 1, Figure 1]. The PPHN + high MAP group had significantly higher mean CVP, OI, and FiO₂ levels than the high MAP group without PPHN (Table 2, Figure 2). Administration of iNO resulted in a significant reduction in the mean CVP values (7 mmHg pre-iNO vs. 4 mmHg post-iNO, *p* = 0.04; Table 3, Figure 3). The initiation of VV-ECMO resulted in a significant increase in the maximum, mean, and minimum CVP values [mean CVP: 8 mmHg [95% CI: 7–10 mmHg] pre-ECMO vs. 13 mmHg [95% CI: 12–14 mmHg] post-ECMO; *p* = 0.03; Table 4, Figure 4].

**Table 1:** Comparison of clinical characteristics, perinatal factors, and physiological parameters between infants with high MAP and those with normal MAP.

Variable	High MAP (*n* = 3, % or IQR)	Normal MAP (*n* = 8, % or IQR)	*p* value
Sex (male)	3 (100)	5 (62.5)	0.49
Birth weight (median, g)	2,784 (2,426–3,408)	2,923 (2,485–3,195)	1
%tile	34.7 (12.5–67.9)	52.2 (32.0–82.5)	0.31
GW	38 (38–41)	37 (36–39)	0.14
C/S	1 (33.3)	5 (62.5)	0.55
PROM	0	2 (25.0)	1
CAM stage 2–3	0	1 (12.5)	1
Funisitis stage 2–3	0	1 (12.5)	1
Abruption	0	3 (37.5)	0.49
Fetal bradycardia	1 (33.3)	3 (37.5)	1
IVH	0	0	–
EOS	0	0	–
Tension pneumothorax	0	0	–
PPHN	0	0	–
Mortality	0	0	–
APS1min	2 (1–4)	2 (1–4)	0.92
APS5min	5 (1–6)	5 (4–7)	0.6
UA pH	7.02 (7.00–7.27)	7.03 (7.0–7.24)	0.8
max CVP [mmHg]	7 (7–8)	7 (5–8)	0.60
mean CVP [mmHg]	6 (5–7)	5 (4–6)	0.03
min CVP [mmHg]	3 (2–4)	4 (3–4)	0.37
HR [bpm]	124 (94–138)	114 (106–133)	0.83
systolic BP [mmHg]	54 (48–55)	58 (51–72)	0.18
mean BP [mmHg]	43 (30–47)	50 (43–57)	0.12
diastolic BP [mmHg]	32 (22–39)	43 (34–44)	0.1
EF [%]	56 (47–60)	64 (48–71)	0.41
OI	12.9 (5.8–14.2)	2.1 (1.8–3.7)	0.01
MAP [cmH_**2**_O]	11.0 (10.0–11.6)	8.6 (8.1–8.9)	0.01
FiO2	0.45 (0.21–0.80)	0.21 (0.21–0.24)	0.1
WQ [ml/kg/day]	80 (80–80)	80 (64–80)	0.24
Adrenaline	0	2 (25.0)	1
DOA	0	0	–
DOB	0	1 (12.5)	1
Chlorpromazine hydrochloride	0	2 (25.0)	1
Fentanyl	3 (100.0)	6 (75.0)	1
Midazolam	0	0	–
Dexmedetomidine	0	1 (12.5)	1
Phenobarbital	0	3 (37.5)	0.4

APS, apgar score; BP, blood pressure; CAM, chorioamnionitis; C/S, cesarean section; CVP, central venous pressure; DOB, dobutamine; DOA, dopamine; EF, ejection fraction; EOS, early-onset sepsis; FiO₂, fraction of inspired oxygen; GW, gestational week; HR, heart rate; IQR, interquartile range; IVH, intraventricular hemorrhage; MAP, mean airway pressure; OI, oxygenation index; PPHN, persistent pulmonary hypertension of the newborn; PROM, premature rupture of membranes; UA pH, umbilical arterial pH; WQ, water quantity.

**Table 2:** Comparison of clinical characteristics and physiological parameters between infants with PPHN and high MAP and those with high MAP without PPHN.

Variable	PPHN + High MAP (*n* = 7, % or IQR)	High MAP (*n* = 3, % or IQR)	*p* value
Sex (male)	4 (57.1)	3 (100)	0.48
Birth weight (median, g)	2,934 (2,804–3,024)	2,784 (2,426–3,408)	0.73
%tile	30.8 (22.4–64.7)	34.7 (12.5–67.9)	0.73
GW	39 (37–40)	38 (38–41)	0.82
C/S	3 (42.9)	1 (33.3)	1
PROM	1 (14.3)	0	1
CAM stage 2–3	1 (14.3)	0	1
Funisitis stage 2–3	1 (14.3)	0	1
Abruption	4 (57.1)	0	0.2
Fetal bradycardia	4 (57.1)	1 (33.3)	1
IVH	0	0	–
EOS	0	0	–
Tension pneumothorax	0	0	–
PPHN	7 (100)	0	0.01
Mortality	0	0	–
APS1min	1 (1–4)	2 (1–4)	0.33
APS5min	2 (2–4)	5 (1–6)	0.35
UA pH	6.92 (6.67–7.01)	7.02 (7.0–7.27)	0.09
max CVP [mmHg]	10 (8–11)	7 (7–8)	0.08
mean CVP [mmHg]	7 (6–8)	6 (5–7)	0.04
min CVP [mmHg]	4 (4–5)	3 (2–4)	0.06
HR [bpm]	132 (122–140)	124 (94–138)	0.43
systolic BP [mmHg]	51 (45–62)	54 (48–55)	0.91
mean BP [mmHg]	42 (31–54)	43 (30–47)	0.65
diastolic BP [mmHg]	35 (25–49)	32 (22–39)	0.49
EF [%]	56 (51–68)	56 (47–60)	0.43
OI	25.4 (17.4–27.2)	12.9 (5.8–14.2)	0.02
MAP [cmH_**2**_O]	11.0 (10–11.6)	11.0 (10–11.2)	0.81
FiO2	1.00 (1–1)	0.45 (0.21–0.80)	0.003
WQ [ml/kg/day]	180 (100–195)	80 (80–80)	0.02
Adrenaline	4 (57.1)	0	0.2
DOA	5 (71.4)	3 (100.0)	1
DOB	4 (57.1)	3 (100.0)	0.2
Chlorpromazine hydrochloride	1 (14.3)	0	1
Fentanyl	6 (85.7)	3 (100.0)	1
Midazolam	1 (14.3)	0	1
Dexmedetomidine	0	1 (33.3)	1
Phenobarbital	2 (28.6)	0	1

APS, apgar score; BP, blood pressure; CAM, chorioamnionitis; C/S, cesarean section; CVP, central venous pressure; Dexmedetomidine, an *α*2-adrenergic receptor agonist; DOB, dobutamine; DOA, dopamine; EF, ejection fraction; EOS, early-onset sepsis; FiO₂, fraction of inspired oxygen; GW, gestational week; HR, heart rate; IQR, interquartile range; IVH, intraventricular hemorrhage; MAP, mean airway pressure; OI, oxygenation index; PPHN, persistent pulmonary hypertension of the newborn; PROM, premature rupture of membranes; UA pH, umbilical arterial pH; WQ, water quantity.

**Table 3:** Changes in CVP before and after initiation of ECMO.

Variable	pre-ECMO (IQR)	post-ECMO (IQR)	*p* value
max CVP [mmHg]	11 (8–11)	15 (13–15)	0.02
mean CVP [mmHg]	8 (7–10)	13 (12–14)	0.03
min CVP [mmHg]	5 (4–8)	11 (10–13)	0.02

CVP, central venous pressure; ECMO, extracorporeal membrane oxygenation; IQR, interquartile range.

**Table 4:** Changes in CVP before and after inhaled NO administration.

Variable	pre-NO (IQR)	post-NO (IQR)	*p* value
max CVP [mmHg]	8 (7–11)	6 (5–7)	0.08
mean CVP [mmHg]	7 (6–8)	4 (3–5)	0.04
min CVP [mmHg]	4 (3–5)	2 (2–3)	0.10

CVP, central venous pressure; IQR, interquartile range; NO, nitric oxide.

## 4 Discussion

These findings suggest a more severe degree of pulmonary hypertension and impaired oxygenation in the PPHN + high MAP group. The significant reduction in CVP following iNO administration may reflect decreased pulmonary vascular resistance and improved right ventricular function.

In contrast, the observed increase in CVP following VV-ECMO initiation may be multifactorial. While our initial explanation suggested elevated right atrial pressure due to return cannula flow, this hypothesis is limited. In VV-ECMO, equal volumes of blood are drained and reinfused per unit time, and CVP is generally not expected to change solely due to circuit flow. More plausible explanations include increased preload from fluid and blood product administration during ECMO initiation, or decreased myocardial contractility due to illness severity or sedation. Although detailed echocardiographic assessments were available for all cases, we have summarized available pre- and post-ECMO echocardiographic data in Tables 1, 2. These data, while limited, suggest no consistent change in left ventricular ejection fraction (LVEF), though other functional indices were not available. Moreover, we cannot exclude the possibility that the return cannula flow itself may have directly influenced CVP readings, particularly if the cannula was positioned in close proximity to the CVP measurement site. This limitation is now acknowledged. The factors influencing CVP fluctuations have long been classified into four primary categories: concepts: vis a tergo (capillary pressure), vis a fronte (cardiac function and resistance), vis a latere (external pressure), and vis a parte interiore (internal volume) (1).

### 4.1 High MAP vs. control

In mechanically ventilated patients, high MAP was associated with higher CVP values in our cohort. This may be due to lung overdistension increasing intrathoracic pressure, which could reduce venous return and raise CVP. However, we did not measure right ventricular afterload or cardiac output, so this mechanism remains speculative (12). As shown in Figure 1, high MAP management significantly increased the mean CVP (6 mmHg in the high MAP group vs. 5 mmHg in the normal MAP group, *p* = 0.03), suggesting that elevated intrathoracic pressure may contribute to impaired venous return and increased CVP.

### 4.2 PPHN + high MAP vs. high MAP

In neonates with both PPHN and high MAP, CVP was higher than in those without PPHN. One possible explanation is that PPHN may increase pulmonary vascular resistance, leading to elevated right ventricular pressures and CVP (Figure 2) (13). However, right ventricular function and pulmonary arterial pressure were not directly assessed in this study.

### 4.3 Pre-post iNO

A significant reduction in CVP was observed after iNO administration. While we did not assess pulmonary vascular resistance or right ventricular function directly, this reduction may reflect improved hemodynamics associated with pulmonary vasodilation. Further studies with comprehensive cardiac assessments are needed to confirm this hypothesis (Figure 3) (14).

### 4.4 Pre-post ECMO

In VV-ECMO, venous blood is drained from the right atrium and oxygenated blood returns to the venous system, effectively replacing the function of the lungs. This allows for the weaning of ventilatory settings and transition to lung-protective ventilation strategies. VV-ECMO may reduce intrathoracic pressure through lung-protective ventilation, which could theoretically improve right ventricular preload and afterload (15). Despite this, our data showed an increase in CVP after ECMO initiation, which may reflect fluid administration, sedation effects, or other hemodynamic shifts. Therefore, while CVP reduction might be expected, our findings suggest a more complex interplay. The observed CVP increase after VV-ECMO initiation is likely due to combined factors, including increased preload from volume resuscitation, myocardial depression due to disease severity or sedation, and possibly direct influence of return cannula flow on the CVP monitoring site. Although return flow in VV-ECMO is generally not expected to increase CVP if the cannula is appropriately positioned, the mechanical interaction between return flow and catheter tip may affect pressure readings, especially in neonates with small vascular structures (Figure 4).

Left ventricular contraction generates the pressure needed to push blood through the capillaries and into the venous system. In this study, LVEF was assessed using M-mode echocardiography; however, this method has inherent limitations. All patients exhibited mildly reduced cardiac motion due to HIE. Nonetheless, initial echocardiography performed after catecholamine and sedative administration showed an EF of ≥45% in all cases, indicating that no patients had heart failure with reduced EF. Furthermore, comparisons between neonates with high MAP and those with normal MAP, as well as those with high MAP with or without PPHN, revealed no significant differences in the EF. CVP measurements were obtained under stable conditions 2–3 h after admission, following catecholamine and sedative administration. Therefore, vis a tergo was considered equivalent across the study groups.

The function of the heart, particularly the right atrium and ventricle, facilitates the return of blood from the venous system. In this study, right atrial and ventricular functions were not specifically evaluated. However, all patients had HIE and were assessed at comparable time points. Furthermore, there were no significant differences in the use of catecholamines (dopamine, dobutamine, and adrenaline), sedatives, or analgesics among the groups. Patients with PPHN were excluded from comparisons between the high and normal MAP groups. Therefore, no significant differences in right heart function were observed, suggesting comparable conditions between the groups. In contrast, comparisons involving high MAP with or without PPHN suggested that the presence of PPHN influences right heart function, contributing to an elevation in CVP.

External pressures, including tissue compression and respiratory factors like intrathoracic or intra-abdominal pressure, can affect venous blood flow and central venous pressure. In this study, all neonates were term births without congenital anomalies or hydrops, and blood cultures obtained at admission were negative, ruling out an infection. Furthermore, there were no significant differences in the use of catecholamines (dopamine, dobutamine, and adrenaline), sedatives, or analgesics among the groups. These factors minimized the impact of vasoconstriction, vasodilation, or inflammation on venous compliance and tone. Patients with diaphragmatic hernias, pneumothorax, meconium aspiration syndrome, intestinal perforation, necrotizing enterocolitis, or abdominal wall defects were excluded, thereby minimizing variations in external pressure from the thoracic or abdominal cavities due to underlying conditions. The effects of intrathoracic pressure resulting from respiratory support were analyzed as potential contributors to CVP by comparing the high MAP and control groups. The findings demonstrated that airway pressure influences CVP levels.

Central venous pressure can also be affected by internal conditions, including the patient's blood volume and fluid status. In this study, water balance and fluid status at the time of admission and measurement were similar across all groups, suggesting that the influence of internal volume on CVP differences was minimal.

## 5 Limitations

This study had several limitations.

First, its retrospective design limited the ability to capture real-time changes in CVP during the acute phases of treatment. Therefore, prospective studies using advanced monitoring systems are warranted.

Second, the relatively small sample size (*n* = 18) limited the statistical power of the study, particularly in subgroup analyses (e.g., high MAP with and without PPHN). Notably, some subgroups—such as the high MAP without PPHN group—included as few as three patients. Such small numbers limit the reliability and reproducibility of intergroup comparisons. In addition, the inclusion period spanned over two decades, during which changes in clinical practice—including ECMO protocols, ventilator settings, sedation strategies, and cardiovascular management—may have introduced unmeasured confounding. These temporal changes should be taken into account when interpreting the results. Thus, future multicenter studies involving larger, more contemporaneous cohorts are needed to validate these findings under consistent clinical conditions.

Third, right heart function was not comprehensively assessed. Although LVEF was evaluated using M-mode echocardiography in all cases, standardized and quantitative assessments of right atrial and right ventricular function were lacking. Because of the retrospective nature of the study and variability in clinical practice over the 22-year study period, echocardiographic assessments beyond EF were not consistently performed or recorded. As such, the influence of right heart dynamics (*vis a fronte*) on CVP could not be directly evaluated.

Fourth, UVC positioning may have affected CVP measurements despite radiographic confirmation. Minor anatomical variations in catheter tip location, even within the correct anatomical zone, could have influenced local pressure readings, particularly in neonates with small vascular structures.

Fifth, although ECMO systems are designed to maintain hemodynamic equilibrium by balancing inflow and outflow, it is possible that return flow from the VV-ECMO cannula directly impacted CVP readings if the cannula tip was located near the measurement site. This mechanical interaction may have transiently increased right atrial pressure or caused turbulent flow at the catheter tip, falsely elevating the CVP. Future studies should consider detailed anatomical evaluation of cannula position and its potential effect on intravascular pressure measurements.

Sixth, the impact of ventilation strategies was not fully analyzed. Although MAP values were compared, we did not assess ventilator modes or lung compliance, both of which may influence intrathoracic pressure and CVP. Future studies should evaluate how different ventilatory modes, such as adaptive ventilation strategies or lower MAP settings, affect CVP dynamics, particularly in neonates with PPHN.

Finally, the generalizability of these findings is limited, as the study focused exclusively on neonates with HIE undergoing therapeutic hypothermia, excluding those with structural anomalies, major malformations, or non-HIE causes of encephalopathy. Broader studies including diverse neonatal populations are necessary to confirm external validity.

## 6 Future directions

Future studies should prioritize multicenter prospective designs with larger sample sizes. Integrating continuous hemodynamic monitoring, advanced echocardiographic evaluation of right heart function, and development of tailored ventilation protocols will further elucidate the dynamic factors influencing CVP in neonates with HIE. Additionally, efforts to refine catheter placement techniques and ECMO flow settings may improve the accuracy and reliability of CVP measurement.

## 7 Summary of results

The high MAP group exhibited significantly higher mean CVP than the normal MAP group. In contrast, the high MAP group with PPHN demonstrated significantly higher mean CVP, OI, and FiO2 levels than the high MAP group without PPHN, indicating more severe pulmonary hypertension and impaired oxygenation.

PPHN was associated with impaired right heart function, reducing venous return to the right atrium and contributing to an elevated CVP.

After the initiation of VV-ECMO, the maximum, mean, and minimum CVP values increased significantly. This increase in CVP was attributed to elevated right atrial pressure caused by return flow from VV-ECMO.

In contrast, iNO therapy reduced pulmonary vascular resistance and improved right ventricular systolic and diastolic function. This enhancement in right heart performance increased venous return to the right atrium and resulted in a decrease in the mean CVP.

## 8 Conclusions

This study demonstrated that CVP monitoring using UVCs is a valuable tool for assessing hemodynamic changes in neonates undergoing therapeutic hypothermia for HIE.

## Data Availability

The raw data supporting the conclusions of this article will be made available by the authors, without undue reservation.
